# Editorial: Women in pharmacology of infectious diseases: 2021

**DOI:** 10.3389/fphar.2022.1038369

**Published:** 2022-10-06

**Authors:** Elena K. Schneider-Futschik, Isabel Spriet, Hong Zhou

**Affiliations:** ^1^ Cystic Fibrosis Pharmacology Laboratory, Department of Biochemistry and Pharmacology, Melbourne University, Melbourne, VIC, Australia; ^2^ Pharmacy Department, University Hospitals Leuven, Leuven and Department of Pharmaceutical and Pharmacological Sciences, KU Leuven – University of Leuven, Leuven, Belgium; ^3^ Key Laboratory of Basic Pharmacology of Ministry of Education and Joint International Research Laboratory of Ethnomedicine of Ministry of Education, Zunyi Medical University, Zunyi, China

**Keywords:** antibiotic resisitance, polymyxins, rifampicin, tuberculosis, cephalosporins, protein binding, escape mechanisms

Presently women continue to be under-represented with less than 30% of researchers worldwide identify as women in science-related fields and science, technology, engineering, and mathematics research. To promote the excellence of women in pharmacology ‘Frontiers in Pharmacology’ offers this special issue to promote the work of women scientists, across all areas of Pharmacology of Infectious Diseases. This Research Topic gathers a collection of four original and review articles that provide novel information regarding different topics of infectious disease at basic, translational, and clinical levels.

The first research article by Chung et al. gives some insights on biological insights into the resistance mechanisms of the multidrug-resistant (MDR) *Klebsiella pneumoniae* against polymyxins. Unfortunately, reports of resistance against lastline antibiotics such as polymyxins (polymyxin B and colistin) are becoming more common. (Moskowitz et al., 2012). Colistin is clinically used as its inactive prodrug colistimethate sodium (Allobawi et al., 2020). The authors in the study herein employed genome-scale metabolic models (GSMMs) to delineate the altered metabolism of New Delhi metallo-β-lactamase- or extended spectrum β-lactamase-producing *Klebsiella pneumoniae* strains. Metabolic network simulation revealed that feeding of 3-phosphoglycerate and ribose 5-phosphate enhanced central carbon metabolism, adenosine triphosphate demand, and energy consumption, which are converged with metabolic disruptions by polymyxin treatment. Furthermore, the authors developed a systematic framework of metabolic analysis and biological assays to facilitate the clinical translation of antibiotic-resistant infection management.

The second research article by Beever et al. investigated the liposomal glutathione (L-GSH) supplementation in combination with the first line antibiotic rifampicin against *Mycobacterium tuberculosis* which causes tuberculosis (TB). According to World Health Organization TB accounts for one of the top 10 causes of mortality with approximately 1.5 million people dying of TB in 2020 alone. Both active tuberculosis (TB) and asymptomatic latent infection (LTBI) causes significant economic and health burdens worldwide. Unfortunately, immunocompromised patients such as individuals with as Type 2 Diabetes Mellitus battle to control *Mycobacterium tuberculosis* infection and are more susceptible to reactivation of latent to active diseases. In a diabetic mouse model, they showed L-GSH supplementation caused a significant reduction in bacterial burden in the lungs, decreased oxidative stress, and increased the production of IFN-γ, TNF-α, IL-17, IL-10, and TGF-β1. Furthermore, the addition of rifampicin with L-GSH yielded better infection levels and oxidative stress than rifampicin alone. Therefore, the supplementation of L-GSH in combination with first-line antibiotic rifampicin may be a promising strategy against *M. tb* infection in individuals with type 2 diabetes.

The third article is a systematic review article by Jongmans et al. nicely summarizing the current knowledge of protein binding of cephalosporins in human body fluids relating to patient characteristics influencing the level of protein binding. Cephalosporins are broad spectrum, beta-lactam antibiotics commonly used in the hospital setting. Changes in plasma protein binding such as in chronic inflammation affect the transportation of endogenous molecules and exogenous drugs (Ward et al., 2022). Cephalosporins such as cefazolin, ceftriaxone, cefpiramide, and cefonicid were found to follow a non-linear pattern in protein binding in serum or plasma. Furthermore, they highlighted that various patient characteristics were associated with low serum albumin concentrations and hence lower protein binding such as critically ill patients, dialysis patients, and patients undergoing cardiopulmonary bypass during surgery. The authors emphasized that therapeutic monitoring is recommended to measure unbound concentrations to optimize antibiotic exposure in patients.

The final contribution is a mini review article by Bhandari et al. dedicated to next-generation approaches needed to tackle antimicrobial resistance. Antibiotic resistance is a major global health challenge and, worryingly, several key pathogens are increasingly resistant to most currently available antibiotics. The authors discussed the emergence of bacterial resistance over time and the various defence and escape mechanisms that emerged in bacterial cells. Furthermore, the authors highlighted new approaches to tackle antimicrobial resistance or advantageous practices in identifying new treatment options.

In conclusion, this issue collates current knowledge on the functional characterization and pharmaceutical targets to tackle antimicrobial resistance. These range from novel insights into new approaches in the development of novel therapies to understanding the mode-of-action of currently available treatments such as polymyxins, rifampicin or cephalosporins. With the breadth of work currently being conducted in the field of combating antimicrobial resistance, the horizon looks optimistic for several approaches to clinical translation of antibiotic-resistant infection management.
